# Bridging fibrosis and function: an integrated mechanistic and therapeutic framework for Peyronie’s disease and related erectile dysfunction

**DOI:** 10.1093/sexmed/qfag070

**Published:** 2026-08-12

**Authors:** Chunlin Wang, Andrea Sansone, Dake Zhu, Shilong Cheng, Yan Zhang, Elena Colonnello, Gilberto Bellia, Claudia Pitrè, Emmanuele A Jannini

**Affiliations:** Section of Endocrinology and Medical Sexology (ENDOSEX), Department of Systems Medicine, University of Rome Tor Vergata, Rome 00133, Italy; Section of Endocrinology and Medical Sexology (ENDOSEX), Department of Systems Medicine, University of Rome Tor Vergata, Rome 00133, Italy; Section of Endocrinology and Medical Sexology (ENDOSEX), Department of Systems Medicine, University of Rome Tor Vergata, Rome 00133, Italy; Section of Endocrinology and Medical Sexology (ENDOSEX), Department of Systems Medicine, University of Rome Tor Vergata, Rome 00133, Italy; Department of Infertility and Sexual Medicine, The Third Affiliated Hospital of Sun Yat-sen University, Guangzhou 510630, China; Section of Endocrinology and Medical Sexology (ENDOSEX), Department of Systems Medicine, University of Rome Tor Vergata, Rome 00133, Italy; IBSA Farmaceutici Italia S.r.l, Medical Affairs, Lodi 26900, Italy; IBSA Farmaceutici Italia S.r.l, Medical Affairs, Lodi 26900, Italy; Chair of Endocrinology and Medical Sexology (ENDOSEX), Department of Biomedicine and Prevention, University of Rome Tor Vergata, Rome 00133, Italy

**Keywords:** Peyronie disease, erectile dysfunction, management, PDE5i, sildenafil, ODF

## Abstract

**Introduction:**

Peyronie’s disease (PD) and erectile dysfunction (ED) are prevalent and frequently coexisting male sexual disorders that share endothelial dysfunction, oxidative stress, and tunical fibrosis as common pathophysiologic pathways. Recognizing their interconnected nature has reframed both conditions within a unified model of penile vascular–fibrotic disease.

**Objectives:**

To synthesize recent advances in the mechanistic, diagnostic, and therapeutic understanding of PD and ED, and to propose an integrated framework for comprehensive sexual medicine care.

**Methods:**

A integrative narrative review of contemporary clinical studies, translational studies, molecular studies and guideline recommendations was conducted to evaluate epidemiologic trends, shared molecular mechanisms, and evolving therapeutic modalities addressing both PD and ED.

**Results:**

Up to 60% of PD patients experience concomitant ED. Both disorders are linked by endothelial nitric oxide synthase dysregulation, oxidative stress, and transforming growth factor-β1–mediated fibrosis. Diagnostic evaluation now integrates penile duplex Doppler ultrasonography, elastography, and psychometric tools to assess vascular, structural, and psychosexual domains. Therapeutic strategies increasingly adopt multimodal approaches: phosphodiesterase-5 inhibitors improve hemodynamics and may attenuate early fibrotic activity; intralesional therapy remains central to nonsurgical management, with collagenase *Clostridium histolyticum* as the established standard for plaque remodeling and adjunct injectables such as verapamil, interferon-α2b, and hyaluronic acid providing additional anti-inflammatory or antifibrotic benefits, particularly in the active phase; and low-intensity shockwave therapy promotes angiogenesis and tissue recovery. Regenerative therapies—including stem cell, exosome, and gene-targeted interventions—offer promising disease-modifying potential. Surgery (plication, grafting, or prosthesis implantation) remains definitive for refractory cases, complemented by psychosexual rehabilitation.

**Conclusions:**

PD and ED represent a shared vascular–fibrotic spectrum requiring integrated, mechanism-driven management. Emerging regenerative and molecular therapies, supported by artificial intelligence–assisted diagnostics and digital health tools, signal a paradigm shift toward personalized, holistic restoration of structure, function, and intimacy.

## Introduction

Peyronie’s disease (PD) and erectile dysfunction (ED) are prevalent male sexual health conditions with significant physical and psychological impacts.^1^^,^^2^ PD is characterized by the development of fibrotic plaques within the tunica albuginea, leading to penile curvature, deformity, pain, and, ultimately, sexual dysfunction.^3^ ED, defined as the persistent inability to achieve or maintain an erection adequate for satisfactory intercourse, affects ~20%–60.1% of men with PD,^4^^,^^5^ a prevalence significantly higher than that observed in the general population. Although the animal models cannot be used with this purpose,^6^ this frequent coexistence suggests more than a coincidental association, reflecting shared pathophysiologic substrates that link structural, vascular, and intrapsychic and relational domains of male sexual health.

Accumulating clinical and molecular evidence now indicates that PD and ED are closely interconnected rather than distinct entities. Repetitive microvascular trauma, oxidative stress, and impaired endothelial nitric oxide (NO) signaling appear to serve as common initiating events, culminating in both tunical fibrosis and cavernosal smooth muscle dysfunction.^7^ These overlapping mechanisms contribute not only to penile deformity and rigidity loss but also to diminished penile hemodynamics and tissue compliance.^7^ Moreover, the chronic pain, altered body image, and anxiety associated with PD often exacerbate or precipitate psychological ED,^8^ further blurring the clinical boundary between the two disorders.

In recent years, advances in molecular biology, regenerative medicine, and imaging have begun to unravel the intricate pathways that bridge these conditions. Novel therapeutic modalities—ranging from phosphodiesterase type 5 inhibitors (PDE5is) and intralesional biologics to low-intensity shockwave therapy and stem cell–based interventions—are redefining treatment paradigms by simultaneously targeting vascular and fibrotic processes.^9^

Against this evolving backdrop, this review aims to synthesize current understanding of the interconnected mechanisms underpinning PD and ED, highlight emerging diagnostic and therapeutic innovations, and propose a conceptual framework that integrates vascular, fibrotic, and psychological dimensions of these overlapping disorders. By doing so, we seek to answer the question: is it possible to advance a more unified model of penile dysfunction that transcends traditional categorical distinctions and guides future research toward comprehensive, mechanism-based management strategies?

## Methods

This integrative narrative review was conducted through a non-systematic search of the literature using PubMed/MEDLINE, Scopus, and Google Scholar databases. The authors reviewed clinical studies, translational investigations, guideline statements, and relevant reviews published primarily between 2000 and 2025 concerning Peyronie’s disease, erectile dysfunction, endothelial dysfunction, fibrosis, regenerative therapies, and psychosexual aspects of male sexual health. Keywords included “Peyronie’s disease,” “erectile dysfunction,” “fibrosis,” “oxidative stress,” “TGF-β,” “penile duplex Doppler ultrasound,” “shockwave therapy,” “stem cells,” and “sexual medicine.” Priority was given to high-quality clinical studies, consensus guidelines, and mechanistic investigations considered most relevant to the integrated pathophysiological and therapeutic framework discussed in this review.^10^

## Epidemiology and clinical overlap

The epidemiological interplay between PD and ED underscores their clinical and pathophysiologic interdependence. Several clinical studies show that 20-60.1% of men with PD report some degree of erectile impairment,^4^^,^^5^ whereas among men presenting with ED, ~8%–10% have clinically or ultrasonographically detectable evidence of PD.^1^ The variability in these figures reflects not only methodological differences but also the dynamic nature of disease progression—whereby tunical fibrosis, vascular insufficiency, and psychorelational factors evolve concurrently. Several studies suggest that more severe penile deformity is associated with greater sexual disability or difficulty with penetrative intercourse in men with PD,^11^^,^^12^ indicating a possible dose–response type relationship between structural alteration and functional impairment. However, the evidence remains inconsistent: some series failed to demonstrate a significant correlation between curvature severity and objective hemodynamic parameters or ED severity.^13^^,^^14^

From a clinical perspective, this overlap presents diagnostic and therapeutic challenges. In some patients, ED precedes the manifestation of PD, while in others, the onset of curvature heralds subsequent erectile decline.^5^ Several observational studies have demonstrated that men with PD may harbor significant vascular dysfunction or subclinical endothelial impairment, and that in broader populations of men with ED, subclinical vascular disease may precede overt ED.^15^ These findings imply that failure to detect subclinical PD (and its associated vascular/fibrotic alterations) in men presenting with ED may delay the initiation of early antifibrotic or vascular-restorative treatments. Conversely, in PD patients, overlooking concomitant vascular insufficiency may contribute to less favorable responses to therapy, especially if treatment is focused only on tunica albuginea remodeling and neglects hemodynamic correction.^16^ Consequently, routine assessment of erectile function in PD patients—and conversely, screening for tunical abnormalities in men with refractory ED—should be considered a best practice in comprehensive sexual medicine care.

## Pathophysiologic interconnections

**Figure 1:**
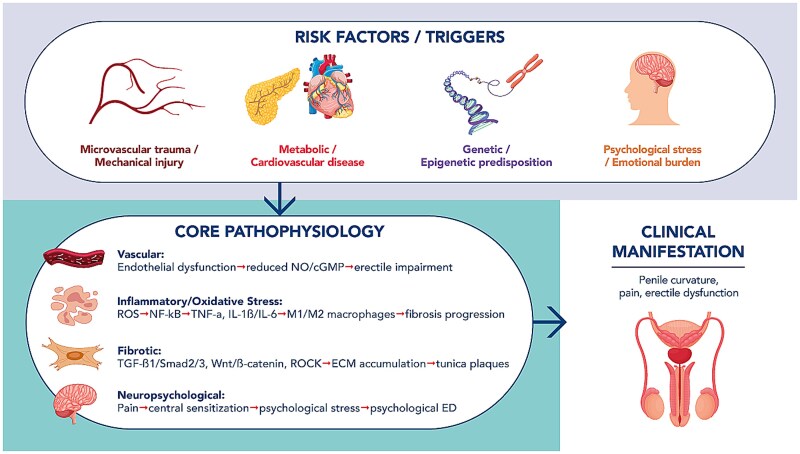
Mechanistic continuum linking PD and ED. This figure summarizes major PD risk factors—including microvascular injury, metabolic disease, genetic susceptibility, and psychological stress—and their convergence on shared vascular, inflammatory, fibrotic, and neuropsychological pathways. Endothelial dysfunction, inflammatory cytokine activity, and TGF-β/ROCK–associated fibrotic signaling are proposed to contribute to plaque development, while pain-related central sensitization and psychological stress may further influence erectile function. Collectively, these interacting processes are thought to underline the co-occurrence of penile curvature, pain, and erectile difficulties in affected individuals.

Growing clinical and molecular evidence suggests that PD and ED represent two interrelated manifestations of a shared penile pathobiology characterized by endothelial injury, immune-mediated inflammation, oxidative stress, and transforming growth factor-β1 (TGF-β1)–driven fibrosis.^17–19^ These mechanisms converge on common downstream events—vascular insufficiency, myofibroblast activation, and extracellular matrix (ECM) remodeling—that culminate in penile deformity and erectile failure. Recognizing this interconnected network provides a conceptual framework for integrated therapeutic targeting of both fibrotic and vasculogenic pathways. Figure 1 (created with BioGDP.com^20^) schematically summarizes this mechanistic continuum.

### Endothelial dysfunction and impaired NO Signaling

Endothelial dysfunction constitutes a pivotal initiating event in both PD and vasculogenic ED.^17^^,^^21^ The penile endothelium regulates corporal hemodynamics via endothelial nitric oxide synthase (eNOS)–mediated release of NO, which activates soluble guanylate cyclase and elevates cyclic guanosine monophosphate (cGMP), promoting smooth muscle relaxation and penile tumescence.^22^ Following penile microtrauma or repetitive mechanical stress, localized inflammation and oxidative stress disrupt endothelial homeostasis.^7^^,^^23^^,^^24^ Increased levels of reactive oxygen species (ROS) scavenge NO and generate peroxynitrite, reducing NO bioavailability and inducing endothelial cell apoptosis.^23^^,^^25^ In PD, TGF-β1 further amplifies oxidative stress and promotes myofibroblast differentiation, linking endothelial injury to progressive fibrosis of the tunica albuginea.^18^^,^^26^ These alterations parallel the endothelial dysfunction observed in vasculogenic ED, where impaired eNOS activity and diminished cGMP signaling compromise cavernosal smooth muscle relaxation and penile perfusion.^15^^,^^22^ Consequently, PD may be viewed not merely as a localized fibrotic disorder but as part of a broader vascular pathology characterized by impaired endothelial signaling and reduced NO–dependent vasodilation.^7^^,^^15^ Genetic polymorphisms affecting eNOS and related regulators further support a shared susceptibility to endothelial injury in both disorders.^21^^,^^27^

Interestingly, recent translational data suggest that PDE5is not only restore NO–cGMP signaling but may also exhibit antifibrotic and anti-inflammatory properties, thereby linking vascular repair to structural modulation.^17^

### Oxidative stress, immune activation, and inflammatory mediators

Oxidative stress and inflammation form the mechanistic bridge connecting endothelial dysfunction and fibrosis in PD and ED.^17^^,^^28^ ROS, largely generated by NADPH oxidase and mitochondrial dysfunction, activate nuclear factor-κB and promote transcription of proinflammatory cytokines—such as tumor necrosis factor-α, interleukin-1β, and interleukin-6—as well as fibrogenic mediators like TGF-β1 and connective tissue growth factor.^19^^,^^29^

Recent work emphasizes the immune contribution, particularly macrophage polarization: M1-type macrophages amplify oxidative and inflammatory signaling, whereas M2 macrophages facilitate aberrant tissue repair, both contributing to tunical plaque formation.^28^ Chronic activation of toll-like receptors and oxidized lipoprotein signaling further sustains the inflammatory milieu.^17^ Collectively, these findings define PD and related ED as immune-metabolic disorders in which oxidative stress and immune activation create a self-perpetuating profibrotic loop.

### Fibrotic Remodeling and ECM dysregulation

Fibrosis constitutes the structural hallmark of PD and a final common pathway of irreversible ED.^24^ The canonical TGF-β1/Smad2/3 axis orchestrates fibroblast-to-myofibroblast transdifferentiation, collagen I/III overproduction, and suppression of matrix metalloproteinase (MMP) activity in favor of tissue inhibitors of metalloproteinases (TIMPs), leading to ECM accumulation and plaque stabilization.^18^^,^^19^

Recent molecular and transcriptomic studies suggest that several signaling cascades—including Wnt/β-catenin, Hedgehog, Yes-associated protein 1/transcriptional coactivator with PDZ-binding motif, phosphoinositide 3-kinase/RAC-α serine/threonine protein kinase, mitogen-activated protein kinase (MAPK), and RAS homologue gene family–associated kinase (ROCK)—may interact synergistically to perpetuate myofibroblast persistence and fibrosis.^18^^,^^26^ Evidence from preclinical models indicates parallel activation of Wnt/β-catenin and p38 MAPK pathways in both PD and diabetic ED, supporting the possibility of a shared fibroinflammatory axis.^18^^,^^29^^,^^30^

Genetic susceptibility may modulate fibroinflammatory responses. Limited familial aggregation studies and candidate gene analyses have identified polymorphisms in TGF-β1, eNOS, and ECM–related genes as potential contributors to both PD and ED.^21^^,^^27^ Available evidence indicates that PD is a heterogeneous disorder influenced by both genetic and environmental factors, where fibrosis-related gene dysregulation—and possibly chromosomal instability—may predispose to aberrant wound healing and plaque formation.^27^ Emerging evidence suggests that epigenetic mechanisms—such as microRNA dysregulation and altered DNA methylation—may contribute to sustained myofibroblast activation and ECM accumulation, potentially linking inherited vulnerability with acquired tissue remodeling.^26^^,^^27^

### Psychological contribution

Although the vascular and fibrotic mechanisms of PD are well characterized, increasing evidence emphasizes the psychological components that significantly influence its presentation and management. Intrapsychic factors, such as emotional distress, disease-related anxiety or depression, and relationship difficulties are reported in a large proportion of patients, often persisting even after surgical correction, indicating that psychological morbidity represents a major component of the overall disease burden.^31–33^ These effects frequently extend to partners, who may experience sexual dissatisfaction and reduced desire that tend to improve following successful treatment, highlighting the importance of partner-inclusive, psychosocially oriented management.^32^^,^^33^

Beyond the interpersonal dimension, psychosexual impact varies across sociocultural contexts, with individuals in sexual minority groups demonstrating greater emotional distress and lower self-esteem, likely due to compounded stigma and relational factors.^34^ Persistent penile pain and altered sensory input during the active phase of PD may influence autonomic and central pain processing pathways. Although direct evidence for sympathetic overactivity or central sensitization in PD is currently lacking, analogous mechanisms observed in chronic pelvic pain and functional sexual disorders provide a plausible conceptual framework.^35^^,^^36^ Within this context, a bidirectional neurovascular–psychological interaction could potentially reinforce both somatic and emotional aspects of the disorder.^37^

## Diagnostic and clinical considerations

Accurate diagnosis and comprehensive evaluation of PD and ED, when co-morbid, are essential for optimal management. Because the two conditions often coexist and share overlapping pathophysiologic mechanisms, clinical assessment must extend beyond isolated symptom appraisal to encompass vascular, structural, and psychologic dimensions. A multidisciplinary and integrative diagnostic approach facilitates not only precise disease staging but also individualized therapeutic planning.

### Clinical evaluation and history taking

A detailed medical and sexual history remains the cornerstone of diagnosis.^38^ In PD, patients commonly report penile curvature, palpable plaques, penile pain during erection, or shortening of penile length.^9^ The clinician should inquire about the onset, progression, and stability of curvature, as these factors distinguish the active inflammatory phase from the chronic stable phase.^9^ A disease-specific instrument, the Peyronie’s Disease Questionnaire (PDQ), is available for the diagnosis and evaluation of PD in both clinical and research settings.^39^ Concurrent assessment of erectile function—using validated instruments such as the International Index of Erectile Function (IIEF-15)—is indispensable, as up to 20-60% of PD patients experience some degree of ED.^5^

For patients presenting with ED, the history should probe potential indicators of PD, such as penile deformity, palpable nodules, or prior trauma during intercourse. Inquiry into cardiovascular risk factors, metabolic comorbidities, and medication history helps elucidate systemic contributors.^40–42^ Psychological screening for anxiety, depression, and relationship strain is also critical, given the bidirectional psychologic reinforcement between PD and ED.^43^ In both disorders, the impact on sexual confidence, partner satisfaction, and quality of life should be explicitly explored, as these psychosocial dimensions often determine treatment priorities and outcomes.^33^^,^^43^

Finally, note that same patients at the initial stages of PD may not have the overt form, fulfilling the clinical definition of ED, but the subclinical ED (SED).^44^ In these patients, we strongly suggest using the Major and Minor Criteria to discover SED^45^ and to treat it by ameliorating the lifestyle and by using PDE5is in selected cases, to avoid the risk of the clinical ED, which may exacerbate the patient’s sufferance.

### Physical examination

Physical examination should be performed in both flaccid and, when feasible, pharmacologically induced erect states.^38^ In PD, palpation of tunical plaques in any penile location remains a key diagnostic finding.^38^ Plaque size, consistency, and mobility may provide indirect information regarding fibrosis extent and disease activity.^46^ Baseline penile length assessment (stretched or erect) may also assist longitudinal follow-up and treatment planning.^47^

For ED assessment, attention should be given to penile anatomy, secondary sexual characteristics, and testicular volume to identify endocrine or congenital abnormalities.^12^^,^^48^ Signs of systemic vascular disease, such as hypertension, further support a vasculogenic etiology.^49^

### Imaging modalities and functional assessment

Only recently the health care providers in the field of sexual medicine took seriously the need to use imaging and to study the erectile function with more than the simple clinical presentation or the use of simple psychometry. While the diffusion of instrumental diagnosis in sexual dysfunction is still a Cinderella in general sexual medicine, in the suspect of ED in PD the attention to the functional assessment is probably more considered in the daily clinical practice.

#### Duplex doppler ultrasound

As suggested by the Italian Society of Andrology and Sexual Medicine (SIAMS) recent guidelines on ED, penile duplex Doppler ultrasonography (PDDU) remains the primary imaging modality for evaluating structural and vascular abnormalities in men with PD and ED.^45^ In fact, dynamic assessment following intracavernosal vasoactive injection allows simultaneous characterization of plaque morphology and penile hemodynamics.^50^^,^^51^ In PD, B-mode and color Doppler imaging facilitate precise visualization of tunical plaques, their echogenicity, calcification, and localization, which are not always appreciable on physical examination alone.^50^ This modality also aids in identifying associated vascular impairment, offering objective data for preoperative assessment and treatment planning.

Ultrasound elastography (UE) has emerged as a potentially useful adjunctive technique for assessing tissue stiffness and plaque characteristics.^52^ By characterizing the biomechanical properties of plaques, UE can detect subclinical fibrosis, monitor disease progression, and evaluate therapeutic response more sensitively than standard ultrasound.^52^ The combined use of PDDU and elastography therefore may bridge the diagnostic gap between fibrotic and hemodynamic dimensions of penile pathology, offering a comprehensive assessment tool for men presenting with ED and suspected PD.^50^^,^^52^ However, current evidence remains limited, and further validation studies are required before routine clinical implementation can be recommended.

#### Magnetic resonance imaging and other modalities

While penile palpation combined with ultrasound remains the primary diagnostic approach for PD, magnetic resonance imaging (MRI) serves as an important adjunct in selected cases. MRI provides excellent soft-tissue contrast and is particularly useful for evaluating plaques located at the penile base, ventrally, or within the septum that may be difficult to detect by ultrasound.^53^^,^^54^ It can also demonstrate periplaque inflammation and delineate the extent of fibrosis, although it is less sensitive than ultrasound for identifying calcifications.^53^^,^^54^ Because of its higher cost and limited additional diagnostic yield, MRI should be reserved for complex or atypical cases, or when malignancy or postoperative changes are suspected.^54^

In addition to MRI, various imaging techniques can assist in the diagnosis and management of PD. Ultrasound and color Doppler ultrasound remain the preferred modalities for identifying plaques, assessing calcifications, and evaluating concomitant vasculogenic ED.^54^^,^^55^ Other emerging methods, such as shear-wave elastography, offer quantitative assessment of penile tissue stiffness and may serve as objective markers for fibrosis progression or therapeutic response in the future.^55^

#### Functional and laboratory assessment

Endocrine evaluation—including serum testosterone, prolactin, and thyroid function tests—is recommended in men with ED to identify hormonal contributors.^56^ In selected cases, nocturnal penile tumescence testing or dynamic infusion cavernosometry may be indicated to further elucidate neurovascular integrity.^57–59^

Laboratory assessment of systemic inflammation or metabolic parameters (eg, C-reactive protein, HbA1c, lipid profile) may reveal underlying risk factors that exacerbate both PD and ED pathogenesis.^60^

### Disease staging and integrated diagnostic models

PD can be broadly categorized into active (acute) and stable (chronic) phases.^38^ The active phase is characterized by penile pain, progressive curvature, and evolving plaque morphology, whereas the stable phase reflects quiescent fibrosis with fixed deformity.^38^ The distinction has significant therapeutic implications—medical therapy is more effective during the active phase, while surgical or mechanical interventions are reserved for stable disease.^3^^,^^38^

For ED, the etiologic classification—vasculogenic, neurogenic, psychological, hormonal, or mixed—remains fundamental to guiding management.^61^ However, in the context of coexisting PD, these categories frequently overlap. For instance, localized fibrosis of the tunica albuginea may impair veno-occlusive function, transforming a primarily structural deformity into a hemodynamic disorder.^62^ Conversely, chronic vascular insufficiency may precipitate ischemia-induced fibrosis within the tunica,^63^ mimicking early PD. These bidirectional interactions blur traditional diagnostic boundaries and underscore the necessity of integrated diagnostic models that evaluate penile structure, blood flow, and psychosexual dynamics concurrently.

An emerging framework posits that PD and ED may represent different phenotypic expressions of a common penile tissue vulnerability, mediated by aberrant wound-healing and fibrotic pathways and modulated by individual risk factors such as diabetes and microtrauma.^26^^,^^29^^,^^41^^,^^64^ Within this framework, penile imaging, endothelial biomarkers, and validated psychometric instruments can be combined into a multidimensional staging system capable of capturing both structural and functional disease components. For example, integrating PDDU parameters (PSV <25 cm/s),^65^ plaque characteristics,^3^ and IIEF scores may allow clinicians to classify patients into distinct subtypes—fibrotic-dominant, vascular-dominant, or mixed—thereby facilitating personalized treatment selection.

### Clinical integration and multidisciplinary management

The diagnostic intersection of PD and ED necessitates a multidisciplinary approach that bridges andrology, urology, sexual medicine, endocrinology, radiology, and psychosexology. The clinician’s role extends beyond identifying physical abnormalities to recognizing the psychosocial impact that curvature and erectile failure impose on patients and their partners.^8^

From a clinical workflow perspective, men presenting with PD should undergo systematic evaluation for erectile function using the IIEF-15 or the masturbation erection index (MEI),^66^ while those with ED—particularly refractory cases—should receive a focused penile examination and, when indicated, imaging to detect occult plaques. Early recognition of subclinical PD in ED patients provides a therapeutic window during which antifibrotic therapies may prevent irreversible structural changes.^7^

**Figure 2:**
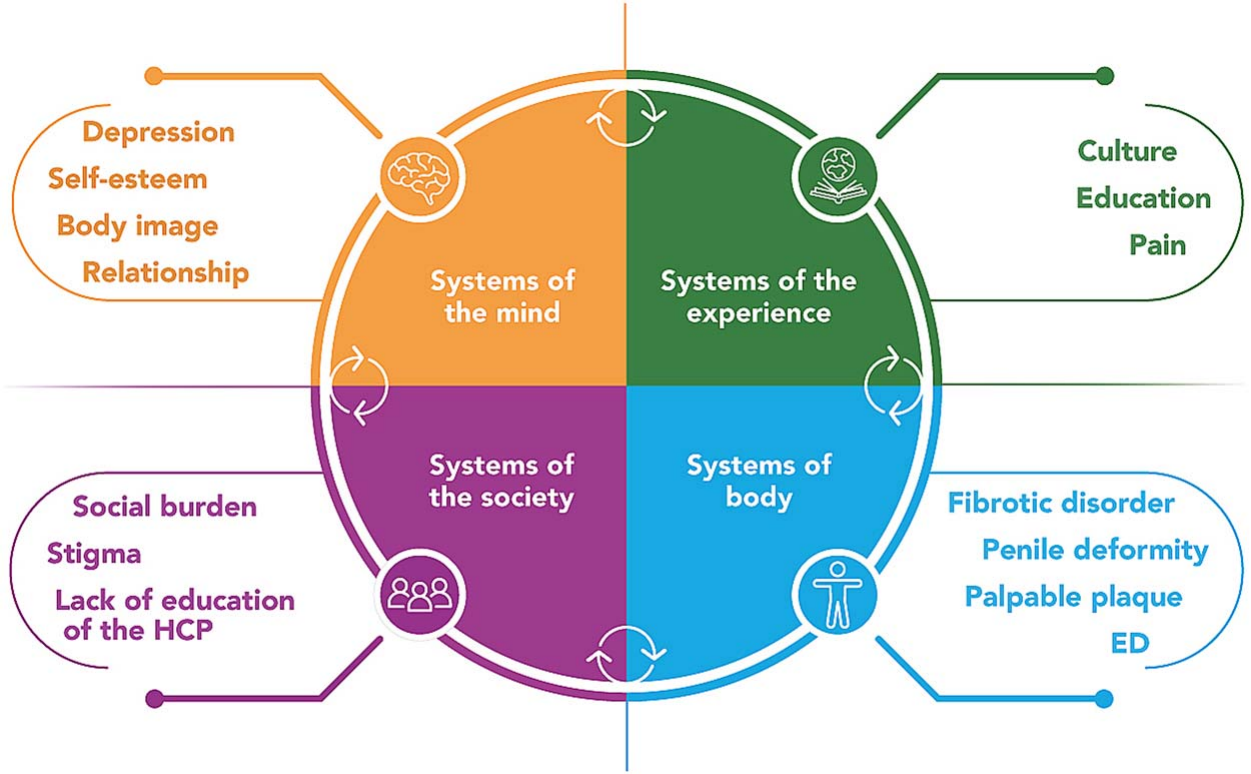
The Peyronie’s disease in the light of the systems sexology paradigm. Systems sexology is an evolution of the biopsychosocial model: Four systems are postulated as developmental producers of health and disease: the systems of mind, the systems of experience, the systems of society, and the systems of the body. The four systems are strictly interrelated and dramatically able to influence each other. Within this perspective, PD and ED emerge not merely as localized penile disorders but as complex conditions shaped by reciprocal biological, psychological, relational, and sociocultural influences. Such a framework may facilitate more comprehensive assessment and individualized therapeutic planning.

Equally important is the inclusion of partner assessment and counseling. Relationship distress and partner avoidance frequently perpetuate anxiety-driven ED.^67^ Evidence suggests that involving partners in diagnostic discussions improves adherence to treatment and overall sexual satisfaction.^32^^,^^68^ The biopsychosocial model^69^ and its recent evolution in the Systems Sexology (SS)^70^ thus serves as the conceptual backbone of integrated PD–ED care, aligning physical correction with psychological restoration. In fact, all four systems (Mind, Experience, Society, and, obviously, Body) of the SS paradigm, as in Figure 2, are dramatically involved not only, as previously conceptualized in ED, but now also in PD.^71^

### Emerging diagnostic innovations

Technological and molecular advances are refining the diagnostic landscape of PD and related ED. Quantitative tools such as three-dimensional penile ultrasound reconstruction and shear-wave elastography offer objective measures of curvature and tissue stiffness.^72^^,^^73^ On the molecular front, endothelial dysfunction–related biomarkers, including asymmetric dimethylarginine^74^ and circulating microRNAs,^75^ may provide insights into the interplay between fibrosis and vascular impairment, but further validation is required.

Artificial intelligence (AI)–assisted image analysis and predictive modeling are also being explored to automate plaque detection and predict disease progression.^76^^,^^77^ These innovations may eventually enable precision diagnostics, linking molecular and imaging signatures to tailored therapeutic algorithms.

Furthermore, the integration of digital health platforms, including mobile-based sexual function tracking and teleconsultation, can facilitate longitudinal monitoring, early detection of relapse, and better adherence to lifestyle or pharmacologic interventions—particularly relevant for younger patients or those with comorbidities.^78^^,^^79^

### Summary and clinical implications

In summary, the diagnosis of PD and ED has progressed from a purely symptom-based approach to a comprehensive, multidimensional evaluation that integrates vascular, fibrotic, endocrine, and psychosocial domains. The convergence of these disorders necessitates an integrative diagnostic paradigm that acknowledges their shared pathophysiology and overlapping clinical manifestations. Clinically, this approach entails the routine assessment of erectile function in all patients with PD and the reciprocal screening for tunical abnormalities in men presenting with ED, as suggested in the Recommendation #14 of the mentioned SIAMS guidelines.^45^ PDDU should be employed as the primary imaging modality to concurrently evaluate plaque morphology and penile hemodynamics. In addition, early identification of endothelial dysfunction and fibrosis-related biomarkers may enable preventive and disease-modifying interventions. Finally, incorporating psychosexual assessment and partner counseling into the diagnostic workflow ensures a holistic understanding of disease burden. By adopting this multidimensional framework, clinicians can more accurately stage disease, tailor treatment strategies, and potentially interrupt the continuum from reversible vascular injury to irreversible fibrosis.

## Therapeutic horizons

Therapeutic management of PD and ED has evolved from symptom alleviation to mechanism-based interventions that concurrently target vascular, fibrotic, and psychologic dimensions. Recognizing these conditions as part of a shared pathophysiologic spectrum underscores the need for integrated, multimodal strategies. The following section summarizes current and emerging therapies for both PD and ED, with a comprehensive overview provided in Table 1.

**Table 1:** Therapeutic Strategies for Peyronie’s Disease (PD) and Erectile Dysfunction (ED).

Therapeutic Category	Representative Interventions	Mechanistic Target/Action	Clinical Application	Evidence Level/Notes
Pharmacologic Therapy	PDE5 inhibitors (sildenafil, tadalafil), pentoxifylline, vitamin E, L-carnitine, CoQ10, testosterone replacement	Enhance NO–cGMP signaling, reduce oxidative stress and fibrosis, restore endothelial function	Early/active PD phase; mild-to-moderate ED	Moderate evidence; PDE5i and pentoxifylline most supported
Approved Intralesional Therapies	Hyaluronic acid (HA), Collagenase *Clostridium histolyticum* (CCH),		Plaque size dramatically decreased with HA Stable PD with curvature 30–90°, preserved erectile function with HA and CCH	High (HA EMA-approved; CCH FDA-approved)
Off label intralesional therapies	Verapamil, Interferon-α2b	Local degradation of collagen I/III, inhibition of fibroblast proliferation, and ECM synthesis		moderate
Association therapies	HA + PDE5 inhibitors		The association is possible.	Efficacy to be demonstrated
Physical/Mechanical Therapy	Penile traction therapy (PTT), Vacuum erection device (VED)	Mechanical stretch promoting collagen remodeling, tissue softening, and penile length preservation	Adjunctive in active or stable PD; post-surgical rehabilitation	Moderate; efficacy depends on adherence
Device-Based/Regenerative Therapy	Low-intensity extracorporeal shockwave therapy (Li-ESWT), Stem cell or exosome-based therapy	Enhances angiogenesis, antifibrotic remodeling, and neurovascular regeneration	PD and vasculogenic ED; early to mid-stage disease	Emerging; promising preclinical and pilot clinical data
Surgical Management	Tunical plication, Plaque incision/grafting, Penile prosthesis implantation (PPI)	Structural correction of curvature, plaque removal, or mechanical rigidity restoration	Severe PD, complex deformity, or refractory ED	High; gold standard for refractory disease
Psychosexual and Rehabilitative Therapy	Cognitive-behavioral therapy (CBT), Partner-based counseling, Penile rehabilitation (VED post-op)	Addresses psychological distress, performance anxiety, and relational factors	Adjunctive to all treatment phases	High; essential for holistic recovery

### Pharmacologic therapy

Pharmacologic approaches remain the first-line management during the early or active phase of PD and for mild-to-moderate ED. The main therapeutic objective is to mitigate inflammation and fibrosis while improving endothelial function and penile hemodynamics.

#### Type 5 phosphodiesterase inhibitors

Beyond their established role in ED, PDE5is can inhibit myofibroblast transformation and exert antifibrotic effects.^80^ Preclinical studies have shown that PDE5is attenuate TGF-β1–induced myofibroblast transformation and collagen synthesis in PD models.^80^ Clinical observational data suggest that continuous PDE5i therapy may provide analgesic benefits and improve erectile function in some patients with active-phase PD^81–83^; evidence for slowing curvature progression is preliminary and inconsistent across studies. Tadalafil, given its prolonged half-life, has been associated with improvements in penile hemodynamic parameters in ED, and early observational reports in acute PD suggest potential benefits for curvature progression and erectile function^83^; however, these findings are preliminary and warrant confirmation in randomized trials. Interesting, a multicenter randomized comparison between the major PDE5is using not only the subjective output but also the objective measurement by PDDU, showed the superiority of sildenafil 100 mg in ameliorating some of the penile flow parameters within the 8-week treatment period.^84^ While this suggests that sildenafil could be considered a potential therapeutic option to treat the PD-related ED, no direct studies exist yet. In particular, it would be particularly interesting to study in the future if the intermediate dose of 75 mg may retain, in this particular subset of patients as it has been found in others, the same clinical power of the maximum 100 mg dose.^85^

#### Pentoxifylline and antioxidant agents

Pentoxifylline has historically been investigated for its antioxidant and antifibrotic properties in PD. It is a non-selective phosphodiesterase inhibitor, that exhibits antioxidant, anti-inflammatory, antifibrotic, and vasorelaxant properties, which may help counteract the pathogenic mechanisms of PD.^86^ Combined with vitamin E, L-carnitine, or coenzyme Q10, it has been hypothesized to reduce plaque progression and curvature in early PD.^86^ However, although some studies suggested possible stabilization of plaque progression or modest curvature improvement, evidence remains inconsistent and largely derived from small or heterogeneous studies. Consequently, its clinical role remains uncertain, and current guideline support is limited. The limited efficacy of traditional antioxidants reflects the complexity of oxidative–fibrotic signaling; future pharmacologic designs may require more specific modulators of ROS-generating pathways or cytokine networks. Finally, and notably, since sildenafil associated with propionyl L-carnitine antioxidant treatment may reduce monocyte activation and markers of endothelial damage in patients with diabetic ED,^87^ the possible pentoxifylline plus sildenafil associations may potentially have a rationale in PD and related ED.

#### Hormonal and anti-inflammatory therapies

Androgen deficiency may exacerbate both endothelial dysfunction and fibrotic remodeling.^88^^,^^89^ Testosterone replacement in hypogonadal men with PD and ED may restore libido and improve response to PDE5is,^90^ though data remain inconclusive.^91^ Similarly, oral anti-inflammatory agents (eg, colchicine, tamoxifen) have shown mixed efficacy and are largely supplanted by intralesional or device-based therapies.^92^

### Intralesional injection therapy

Some intralesional injections of various substances attempted in the past to directly act on the PD lesion, as the intralesional verapamil injection introduced by Levine and coll. in 1994 with the aim to prevent, reduce, slow, and reverse plaque formation and progression.^93^ However, the clinical output was not always successful. Intralesional therapy directly targets the fibrotic plaque and remains the cornerstone of nonsurgical PD management.^92^

#### Collagenase clostridium histolyticum

CCH selectively cleaves type I and III collagen, the principal components of PD plaques.^94^ Multicenter randomized trials (IMPRESS I and II) demonstrated significant curvature reduction (average 34%) and improved sexual function.^95^ Beyond structural correction, secondary analyses reveal modest improvements in IIEF scores, indicating a psychosexual and possibly hemodynamic benefit.^94^ CCH therapy is most effective in men with stable plaques, curvature between 30° and 60°, and preserved erectile function.^94^^,^^96^

Interestingly, a combined treatment trial with CCH, vacuum erection device (see below), and a chronic assumption of a PDE5i on ~100 PD patients, has been shown satisfactory and well tolerable in more than 80% of the sample.^97^ This suggests that multiple treatments for a complex disease, such as PD, may dramatically improve final clinical outcomes.

#### Verapamil and interferon-α2b

Verapamil, a calcium-channel blocker, inhibits fibroblast proliferation and ECM synthesis, while interferon-α2b reduces fibroblast activity and collagen production.^92^^,^^98^ Clinical trials show moderate curvature improvement and pain reduction, though long-term effects on erectile function remain limited.^99^ Despite variable efficacy, intralesional therapy remains valuable for patients seeking minimally invasive options or for those unfit for surgery. However, the compounds described in this and previous paragraphs may produce mild to severe adverse events^100^^,^^101^ (Table 2). Thus, other injective approaches have been studied.

**Table 2:** Potential occurrence of mild to severe adverse events in classic intralesional injection therapies.

Compound	Side effects
Verapamil	penile bruising, dizziness, nausea, and pain at the injection site
Collagenase	penile ecchymosis, swelling, pain, and corporal rupture
Interferon alfa-2b	sinusitis, flulike symptoms, and minor penile swelling

#### Hyaluronic acid

Being the healing process of PD believed to be impeded by trauma or microtrauma, which causes the formation of fibrous scar tissue, its pathophysiology is the inflammation observed in the acute phase, caused by free radicals. It is noteworthy that at this stage of the disease, the plaque on the tunica albuginea of ​​the penis has not yet fully calcified. For this reason, the strategy to early treat the plaque is to be considered one of the most effective.

Hyaluronic acid (HA) is a glycosaminoglycan and a major component of the extra-cellular matrix which is largely prescribed in several fields of medicine for its peculiar ability to reduce the synthesis of pro-inflammatory proteins and its activity against the oxidative stress, characteristic of many degenerative illnesses.^102^^,^^103^ In fact, HA plays a pivotal role in maintaining the major biomechanical and trophic characteristics of the connective tissues of mammals as well as in some peculiar roles such as that in the matrix surrounding the mammalian oocytes.^104^^,^^105^ Interestingly, it has also hypoallergenic properties and, when injected, it is physiologically metabolized and then reabsorbed. Notably, at physiological pH, HA is present in high concentrations in the normal tunica albuginea where maintains hydration, turgidity, plasticity, and viscosity in the amorphous connective matrix.^106^

For all these reasons, HA has emerged as an intralesional therapy for the early stages of PD.^107^ In fact, thanks to its antioxidant and antifibrotic effects, it reduces plaque-related symptoms and limits growth. HA is marketed as a gel, containing as the main component HA sodium salt to be administered by intralesional injections. The physical characteristics of HA make it a hydrophilic agent in a highly polarized environment, maintaining hydration, turgor, plasticity, and viscosity in the amorphous connective tissue matrix. Moreover, HA may express in PD its intrinsic antioxidant effects producing to a reduction of plaque related symptoms coupled with an antifibrotic effects limiting plaque growth. Multiple studies support these claims, having evaluated HA’s efficacy, particularly in the active or acute phase of PD, demonstrating improvements in key clinical outcomes with a minimally invasive approach and minimal side effects.

Intralesional HA injections have consistently shown significant reductions in plaque size and penile curvature. In one study involving 83 PD patients treated with 30 injections of 20 mg HA over 6 months, plaque size decreased by 93.7% (*P* < 0.0001) and penile curvature improved by 9.01° (*P* < 0.0001) at 12-month follow-up, with results stable at 24 months.^108^ Another multicenter pilot study of 65 patients receiving weekly 16 mg/2 mL HA injections for 10 weeks reported a reduction in plaque size from 10 mm to 8 mm (*P* < 0.0001) and curvature from 30° to 20° (*P* < 0.0001), preferably in the early phase of the disease.^100^ In a stable-phase PD cohort of 62 patients treated with three HA injections over 6 weeks combined with vacuum device therapy, stretching, and modeling, curvature decreased from 52.7° to 40.3° (*P* < 0.001), with 80.6% reporting subjective improvement.^109^ While it could be argued that the minimum clinically important difference (MCID) for penile curvature should be around 20°, as in the IMPRESS trials,^95^ the aforementioned reports on HA injections found that a penile curvature improvement of around 10° is also positively evaluated by patients.

The EAU guidelines on Sexual and Reproductive Health mention HA as intralesional treatments of PD (Subchapter 8.2.3.1.2).^110^ Comparative studies support the strength of this therapeutic strategy. A randomized trial of 42 patients in acute-phase PD found that weekly HA (16 mg/2 mL) for 12 weeks reduced curvature by 4.60° (*P* < 0.001), while verapamil showed no significant change, with HA also yielding better patient global impression of improvement (PGI-I) scores (4.0 vs. 2.0; *P* < 0.05).^111^ In a larger prospective study of 244 patients, HA reduced curvature by 9.50° after 8 weekly injections, compared to 4.50° with verapamil (*P* < 0.01).^112^ Another randomized trial in acute-phase PD (n = 42) showed HA decreasing curvature from 34.1° to 24.7° (*P* = 0.005), outperforming verapamil’s reduction from 36.2° to 30.8° (*P* = 0.047), with a significant between-group difference (*P* = 0.038).^113^ Long-term follow-up in 244 patients confirmed HA’s curvature improvement (median 25.0° at 3 months, stable at 1 and 2 years).^7^

Finally, and interestingly, as per the European Medical Devices Regulation (MDR),^114^ the Consultation Procedure for Clinical Evaluation (CECP) assigned the HA device marked as *Perovial* a “High Level of Novelty” classification regarding its suitability and applicability in the management of PD in the acute phase.^115^ The CECP is a regulatory process, primarily under the MDR, where an expert panel reviews the clinical evidence for certain medical devices before a notified body grants certification. This recognition reflects the degree of innovation and the potential positive impact the device can have on the treatment of this condition, placing it at the highest level of novelty compared to existing options.

#### Hyaluronic acid for erectile dysfunction and its association with other specific treatments

It has been demonstrated that HA therapy enhances erectile function in treated PD patients and reduces pain. In a first study, IIEF scores increased by 3.8 points (21.1%; *P* < 0.0001) at 12 months, stable at 24 months.^108^ In a pilot study it has been also noted IIEF-5 scores rising from 20 to 21 (*P* < 0.0001), with ED rates dropping from 55% to 40%, and visual analog scale (VAS) for sexual satisfaction improving from 6 to 8 (*P* < 0.0001), alongside 69% overall PGI-I improvement.^100^ In stable-phase PD, PDQ domains improved significantly (all *P* ≤ 0.01).^109^ Comparisons with verapamil showed HA yielding greater IIEF-15 improvements (1.0 vs. 0.0; *P* < 0.05) and VAS reductions (-4.0 vs. -1.0; *P* < 0.05),^112^ with superior penile pain reduction and patient satisfaction in acute-phase PD.^113^

Combination therapies amplify benefits. HA combined with autologous platelet-rich plasma (PRP) in 90 patients (8 mL injections repeated 4 times over 2 months) reduced curvature by 39.65% (*P* < 0.001) and tunica albuginea thickening by 26.23% (*P* < 0.001), with 73.3% achieving satisfactory improvement, including easier sexual intercourse in 46.7%.^116^ Oral HA plus intralesional injections in a phase III trial (n = 81) reduced curvature by 7.8° (*P* < 0.001) versus 4.1° with intralesional HA alone, with better IIEF-5 (+4 vs. +2; *P* < 0.001) and PGI-I (3 vs. 1; *P* < 0.001).^117^

Although direct clinical trials specifically evaluating the combination of HA injections and the new and promising sildenafil oral dispersible film (ODF) for PD are limited, emerging evidence suggests potential synergistic benefits in managing both the fibrotic aspects and associated ED. Sildenafil ODF, a rapidly dissolving form that enhances bioavailability for ED treatment,^118^ can be particularly efficient in alleviating penile pain, shrink plaques, and improving IIEF scores in PD patients. Notably, studies on combination therapies, such as sildenafil paired with CCH, demonstrate superior outcomes in curvature correction and ED management compared to monotherapy, implying that integrating sildenafil ODF with HA could offer a comprehensive, non-surgical approach by targeting fibrosis through HA and vascular/erectile symptoms via sildenafil, though further randomized trials are needed to confirm efficacy and safety. Long-term data at 2 years showed sustained VAS improvements (-4.0; *P* < 0.01) with HA, though not significantly different from verapamil beyond 3 months.^119^

**Figure 3:**
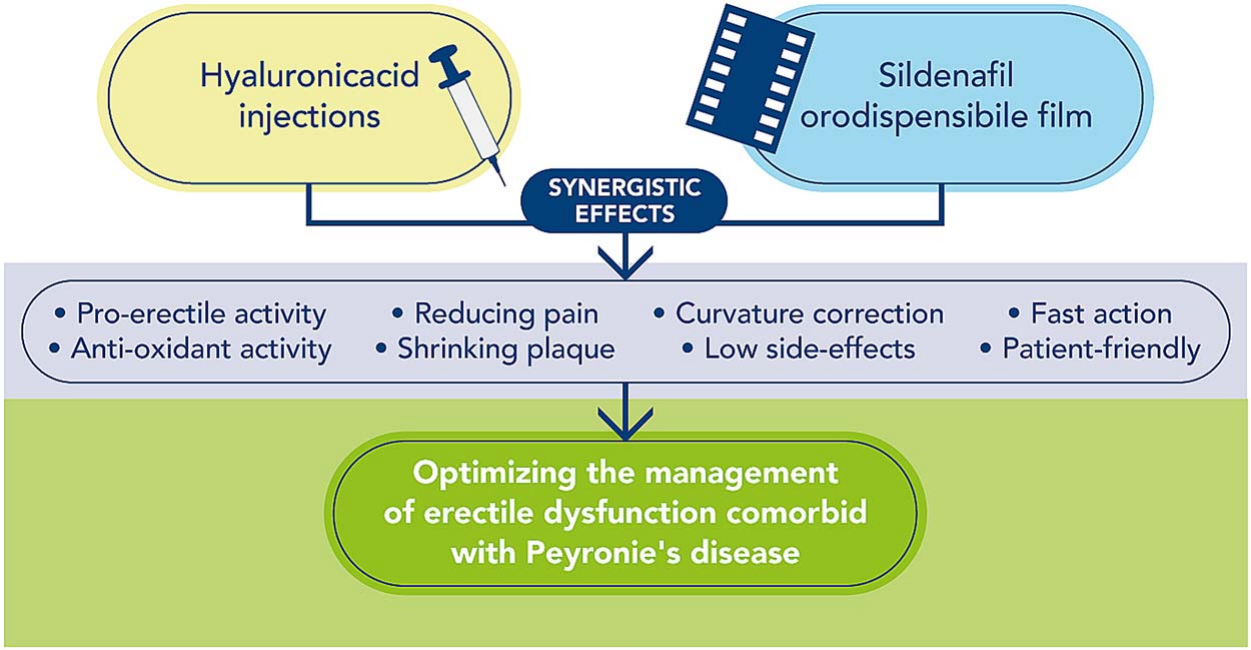
Rationale for the possible co-administration of hyaluronic acid and type 5 phosphodiesterase inhibitors, such as sildenafil orodispersibile film, in Peyronie’s disease comorbid with erectile dysfunction. The two treatments share several mechanisms of action, and a synergic effect could be hypothesized.

In summary, HA’s ability to retain an extremely high amount of water softens plaque on the tunica albuginea of ​​the penis and facilitates proper healing, counteracting scar progression. HA injections are well-tolerated, easy to perform, and associated with no major side effects across studies.^100^^,^^108^^,^^109^^,^^116^ They are particularly indicated in early/active-phase PD,^100^^,^^112^^,^^113^ though potentially beneficial in the stable phase.^109^ Younger patients may achieve better results.^116^ Overall, HA offers a reliable conservative option, potentially stabilizing PD, reducing surgical needs, and improving quality of life without disease worsening over time.^113^^,^^119^ The association of HA with sildenafil, particularly in its innovative and patient-friendly ODF formulation, seems a promising new avenue of research in subjects with PD and related ED (Figure 3).

#### Electromotive drug administration

Electromotive drug administration (EMDA) is a method of increasing transport of drugs across barriers by using of an electric current. In patients with PD, EMDA of verapamil into the tunica albuginea has been found to produce measurable drug levels in plaque tissue. Some clinical studies using different methods showed that EMDA of verapamil and dexamethasone is a safe and effective alternative approach to PD, reducing plaque volume, penile deviation, and pain, and can contribute to the improvement in sexual functioning.^120^^,^^121^

### Physical and mechanical therapies

Given the anatomic nature of the PD, some physical and mechanical therapies have been proposed. They could be used alone or, in the best settings, along with the pharmacological interventions and counseling.

#### Penile traction therapy

Penile traction therapy (PTT) applies controlled mechanical stretching to promote tissue remodeling and potentially reduce curvature.^122^ Several commercially available devices, including RestoreX and other traction systems, such as SizeGenetics, PeniMaster Pro, have demonstrated modest improvements in curvature and penile length in selected patients.^123^ Given that RestoreX has published Level 1b evidence, outcomes vary considerably according to adherence, duration of daily use, and disease phase.^124^ Current evidence does not consistently demonstrate significant improvement in erectile function. PTT exerts controlled mechanical stretch on the penile shaft, promoting cellular mechanotransduction, collagen remodeling, and tissue softening.^125^ Anecdotal data suggest the need to continue to use PDE5is in stretched patients.

#### Low-intensity extracorporeal shockwave therapy

Low-intensity extracorporeal shockwave therapy (Li-ESWT) has been explored as a minimally invasive treatment for both PD and vasculogenic ED. In ED, some studies report temporary improvement in erectile function and responsiveness to PDE5i in selected patient populations, although long-term durability remains uncertain. Preclinical studies demonstrate that Li-ESWT stimulates angiogenesis and neural regeneration—mechanisms associated with upregulation of VEGF and hypoxia-inducible factor-1 alpha (HIF-1α) and recruitment of endothelial progenitor cells—and may modulate profibrotic TGF-β1/Smad signaling to favor antifibrotic remodeling.^126–128^ In PD, available evidence remains heterogeneous and inconsistent. Current guideline recommendations primarily support Li-ESWT for pain reduction during the active phase rather than for reliable curvature correction or disease modification. Additional standardized randomized trials are required to clarify its long-term efficacy and optimal indications.

A meta-analysis claims that Li-ESWT may improve erectile function in men with vasculogenic ED, with increases in IIEF scores and penile hemodynamic parameters.^129^ In PD, some data suggest that Li-ESWT may reduce plaque size, decrease penile curvature, and alleviate pain, though transient improvements in sexual function remain inconsistent across studies.^130^

Despite heterogeneity in energy flux densities and treatment protocols, current findings support Li-ESWT as a safe, minimally invasive therapy that targets the shared vascular–fibrotic axis underlying PD and ED. However, current international guidelines do not recommend Li-ESWT for PD as a first- or second-line treatment with strong recommendation levels. In particular, the American Urological Association guidelines suggest not using ESWT to reduce penile curvature or plaque size. While clinicians may offer it to improve penile pain.^131^ This demonstrates that further standardized, large-scale randomized trials are warranted to define optimal energy parameters, dosing schedules, and patient selection criteria.^130^

### Regenerative and emerging therapies

Advances in regenerative medicine have opened new horizons for disease modification rather than mere symptom control. A recent review of the literature by Kaltsas and Hatzichristou advocates a role for a “regenerative sexual medicine” as an ongoing, yet largely futuristic, paradigm, shifting from empiric biologic injections toward anatomically grounded, evidence-based, exposure-controlled therapies for both PD and ED.^132^

#### Stem cell and exosome-based therapies

Recent developments in regenerative medicine suggest that stem cell–based interventions may offer restorative potential for both PD and ED. Preclinical data have demonstrated that adipose-derived stem cells (ADSCs) exert antifibrotic, angiogenic, and neurotrophic effects via paracrine signaling, secreting trophic factors such as VEGF, insulin-like growth factor-1 (IGF-1), and stromal-derived factor-1 (SDF-1)—as summarized in recent reviews on the cellular mechanisms of ADSC therapy for ED.^133^ These paracrine mediators contribute to inflammation modulation, ECM remodeling, and neurovascular regeneration. Experimental PD models have confirmed that local ADSC administration can prevent or reverse tunical fibrosis, improve erectile hemodynamics, and restore penile structure by enhancing MMP activity and reducing TIMP expression.^134^ A systematic review of the PD inflammatory microenvironment also highlighted that stem cell–based therapy appears most beneficial during the acute, inflammatory phase of the disease when fibrosis remains reversible.^135^

Clinical evidence remains preliminary but encouraging. In pilot human studies using autologous adipose-derived regenerative cells (ADRCs), the procedures were well tolerated, and many participants reported reduced distress and subjective improvement in penile shape, though no significant change in penile curvature was observed.^136^ Narrative reviews of restorative therapies have likewise concluded that while preclinical and early clinical findings suggest potential benefits of stem cells for reducing curvature and plaque size, the American Urological Association, European Association of Urology and the SIAMS continue to regard such therapies as experimental pending stronger evidence.^137^

Parallel to cell-based approaches, ADSC-derived exosomes are emerging as a promising cell-free therapeutic alternative. These extracellular vesicles carry cytokines, growth factors, and regulatory microRNAs that reproduce many of the regenerative and immunomodulatory actions of stem cells without the risks associated with live-cell transplantation.^138^ Building on this concept, next-generation strategies—such as hydrogel delivery systems, magnetic guidance, or genetic enhancement of ADSCs—are being developed to enhance local retention and therapeutic potency.^133^ Overall, current evidence indicates that stem cell and exosome-based therapies provide a biologically plausible and safe foundation for future restorative treatment of PD and ED, though large, well-controlled trials with standardized protocols and long-term follow-up remain necessary to confirm durable efficacy and guide clinical translation.

#### Gene and Molecular targeted therapies

Emerging evidence highlights that PD pathogenesis involves complex interactions among genetic factors, cellular signaling pathways, and environmental triggers, such as microtrauma-induced inflammation.^26^^,^^139^ Key molecular mediators implicated in PD include the TGF-β/Smad signaling pathway, which promotes fibroblast activation and ECM deposition, and proteins such as Kindlin-2, which modulate Smad3 phosphorylation and amplify TGF-β1-mediated fibrotic responses.^140^

Single-cell spatial transcriptomic analysis in rat models of PD revealed distinct heterogeneity among fibroblast and immune cell populations within the tunica albuginea, identifying dysregulation of ribosomal proteins, the unfolded protein response, and the Egfr smrte pathway as potential drivers of fibroblast activation and ECM deposition, contributing to penile fibrosis.^139^ These insights suggest that precise modulation of these pathways may serve as a therapeutic avenue. Moreover, molecular targets such as Lumican (Lum), identified as a regulator of TGF-β signaling and matrix deposition, offer additional candidate targets for pharmacological intervention.^139^

Based on these mechanistic insights, gene- and molecular-targeted therapies are being explored to prevent or reverse plaque formation in PD. Strategies under investigation include small molecule inhibitors or siRNA targeting TGF-β/Smad3 signaling, modulation of Kindlin-2 activity, and interventions designed to mitigate endoplasmic reticulum stress within activated fibroblasts.^139^^,^^140^ These approaches aim to halt or reverse fibrotic remodeling, reduce penile curvature, and preserve erectile function. Integrating genomics, molecular pathway analysis, and phenotypic characterization may enable personalized therapeutic strategies, allowing targeted interventions based on individual patient molecular profiles.^26^

### Surgical management

Surgical intervention remains the definitive treatment for men with severe penile deformity, refractory ED, or both. The choice of procedure depends on curvature severity, erectile capacity, and patient expectations.

#### Tunical plication and plaque incision/grafting

Classic tunical shortening procedures include the Nesbit technique and Yachia corporoplasty, both of which remain established surgical options for correcting simple penile curvature in patients with preserved erectile rigidity. These approaches provide reliable straightening with relatively low morbidity.^141^ The technique—later pioneered by Essed and Schroeder and refined by Lue’s “16-dot” modification—achieves reliable penile straightening without neurovascular dissection or tunical incision.^142^ However, penile shortening remains its major limitation, reported in up to 90% of cases, and may be particularly problematic in men with severe curvature or preexisting erectile dysfunction.^143^ Other drawbacks include palpable sutures, sensory changes, and postoperative erectile or sexual dysfunction in a minority of patients.^143^ Despite these limitations, long-term follow-up studies demonstrate durable cosmetic and functional satisfaction in appropriately selected patients, reaffirming plication as the preferred option for patients with simple curvature and preserved erectile rigidity.^143^ Recent comparative analyses have further supported these findings, showing that plication yields high satisfaction rates with fewer complications compared to tunical lengthening techniques, albeit at the expense of penile length.^144^

By contrast, plaque incision or excision with grafting is designed to restore penile length and correct more complex deformities by lengthening the contracted (concave) side of curvature. Autologous venous grafts—particularly saphenous vein segments—offer favorable elasticity, physiologic compatibility, and low donor-site morbidity.^143^ Early postoperative outcomes demonstrate high rates of penile straightening (up to 96%) and initial satisfaction exceeding 85%.^143^ Nonetheless, long-term follow-up data reveal a gradual decline in satisfaction due to recurrent curvature, penile shortening, and *de novo* erectile dysfunction, raising concerns about durability.^143^ Recent studies comparing various graft materials—including saphenous vein, buccal mucosa, bovine pericardium, and testicular tunica vaginalis—have shown comparable efficacy in penile straightening, length restoration, and erectile function, with no clear superiority among graft types.^145–147^ These grafting techniques, while more technically demanding and associated with longer operative times, remain valuable for patients with severe or complex curvature where tunical lengthening is required to achieve functional correction. Comparative series indicate no significant difference in overall satisfaction or curvature correction between grafting and plication, though grafting carries a higher risk of sensory loss and erectile rigidity decline.^144^^,^^148^ Consequently, tunical plication is generally favored for patients with simple deformities and preserved erectile function, whereas plaque incision and grafting are reserved for more severe or complex cases requiring structural length restoration.

#### Penile prosthesis implantation

The Penile Prosthesis Implantation (PPI), particularly inflatable penile prosthesis (IPP), remains the gold standard surgical option for PD with refractory ED.^149^^,^^150^ It may improve penile rigidity and corrects curvature, while adjunctive maneuvers such as modeling, plication, plaque incision/excision, and grafting are applied when residual curvature exceeds 30° or significant deformity exists.^149–151^ Systematic reviews confirm that PPI with grafting is safe and effective, yielding satisfaction rates of 80–100% and average penile length gains of around 2–3 cm, regardless of graft material.^149^^,^^152^ Furthermore, PPI can also serve as a first-line surgical approach even in PD patients without ED, with similar safety and satisfaction outcomes exceeding 85%.^153^

For severe or complex PD cases, additional straightening procedures may be required during prosthesis implantation. Grafting techniques are preferred for patients with marked penile shortening, while the collagen fleece–based penile implant in combination with the sealing (PICS) technique effectively corrects residual curvature and shortens operative time.^149^^,^^151^^,^^154^ Patient and partner satisfaction remain high overall, though those requiring incision or grafting report slightly lower scores due to penile shortening or glans hypoesthesia.^155^ Collectively, evidence supports PPI as a durable and highly satisfactory solution for PD with or without ED, though further randomized studies are needed to optimize graft selection and refine reconstructive strategies.^149–152^^,^^154^^,^^155^

### Psychosexual and rehabilitative strategies

Because psychological distress, anxiety, and relational conflict frequently perpetuate ED and amplify PD’s impact,^8^ psychosexual interventions should accompany biomedical treatment. Cognitive-behavioral therapy (CBT), sexual counseling, and partner-based therapy enhance coping, improve adherence to mechanical or pharmacologic regimens, and restore intimacy.

Adjunctive penile rehabilitation—including pre- and postoperative vacuum erection device (VED) therapy—has been shown to enhance surgical outcomes and preserve penile length.^156^

### Summary and future outlook

The therapeutic landscape of PD and ED is rapidly transitioning from segregated management toward integrated, multimodal, and regenerative paradigms. Pharmacologic agents address the inflammatory and endothelial phases; intralesional and physical therapies remodel fibrotic tissue; and regenerative approaches may promise in future true disease modification. Surgery remains the cornerstone for refractory cases, while psychosexual care provides the essential humanistic dimension of recovery.

Future directions emphasize early intervention during the active phase to prevent irreversible fibrosis, alongside the use of combination therapy integrating PDE5isand regenerative agents such as HA. In addition, molecularly targeted and gene-based antifibrotic treatments hold promise for more precise and durable outcomes. The incorporation of digital health technologies and AI-assisted precision planning further enhances individualized care. Together, these advances move the field closer to a holistic model of penile restoration, addressing not only anatomical form and physiological function but also the emotional and relational dimensions of male sexual health.

## Emerging directions and future perspectives

Emerging research increasingly supports the concept that PD and ED share overlapping vascular, inflammatory, and fibrotic mechanisms. Advances in molecular biology, regenerative medicine, and imaging technologies may facilitate more individualized approaches to diagnosis and treatment in the future. However, many proposed regenerative and molecular therapies remain experimental, and robust clinical validation is still lacking.

Future priorities include earlier identification of patients at risk of progressive fibrosis, development of standardized outcome measures, and large randomized trials evaluating multimodal treatment strategies. Integrating psychosexual support with medical and surgical management remains essential for comprehensive care.”

## Limitations

The integrated, narrative (non-systematic) design of this review could be associated with a relatively high risk of selection bias; however, we sought to reduce this risk by also evaluating observational and small-sample studies in PD research. The reliance on surrogate endpoints (curvature degrees, IIEF points) rather than patient-relevant outcomes in many cited trials may further call for stronger urological research. The lack of long-term follow-up data for most emerging therapies discussed suggests how young, but promising, is the field of the SS of the association of ED + PD. In fact, despite significant advances in the understanding and management of PD and related ED, important limitations remain. Some of the current evidence derives from observational studies, small cohorts, or heterogeneous therapeutic protocols, limiting comparability across studies. For several conservative and regenerative approaches—including traction therapy, Li-ESWT, stem cell interventions, and some intralesional agents—long-term efficacy and disease-modifying potential remain insufficiently established. Moreover, improvements in surrogate outcomes or curvature measurements do not always translate into clinically meaningful functional benefits or patient satisfaction. The overall limited success of several physical and medical treatments in altering the natural history of PD - which, in some cases, progresses without remission regardless of treatment – advocates for more pre-clinical and clinical studies. Consequently, future basic and large-scale randomized controlled trials with standardized outcome measures are needed to better define optimal patient selection, therapeutic timing, and durable clinical benefit. A holistic perspective, such as the one we provided here, should help with these efforts.

## Conclusion

In conclusion, by examining the evidence, it is possible to advance a more unified model of penile dysfunction. In fact, PD and ED frequently coexist and share common endothelial, inflammatory, fibrotic, and psychosexual mechanisms. Recognizing this overlap supports a more integrated diagnostic and therapeutic approach that considers both structural and functional dimensions of male sexual health.

Although substantial progress has been achieved in understanding disease mechanisms and expanding therapeutic options, many conservative and regenerative interventions still require stronger evidence before widespread adoption. Multidisciplinary management combining medical, surgical, and psychosexual strategies remains essential to optimize patient outcomes and quality of life. Looking forward, the integration of biologic insight, technological precision, and humanistic care defines the emerging paradigm of sexual medicine shaped by the new SS. In this model, PD and ED are addressed not as isolated conditions, but as dynamic disorders that may progress without timely management, yet remain responsive to early detection, multimodal therapy, and holistic rehabilitation. Such a vision moves beyond the restoration of penile form and function—it aspires to reestablish the full spectrum of male sexual health and well-being.
